# Six month lower-leg mechanical tactile sensory stimulation alters functional network connectivity associated with improved gait in older adults with peripheral neuropathy – A pilot study

**DOI:** 10.3389/fnagi.2022.1027242

**Published:** 2022-11-03

**Authors:** Chun Liang Hsu, Brad Manor, Ikechkwu Iloputaife, Lars I. E. Oddsson, Lewis Lipsitz

**Affiliations:** ^1^Hebrew SeniorLife, Hinda and Arthur Marcus Institute for Aging Research, Roslindale, MA, United States; ^2^Harvard Medical School, Boston, MA, United States; ^3^Division of Gerontology, Beth Israel Deaconess Medical Center, Boston, MA, United States; ^4^Division of Rehabilitation Science, Department of Rehabilitation Medicine, Medical School, University of Minnesota, Minneapolis, MN, United States; ^5^RxFunction Inc., Eden Prairie, MN, United States

**Keywords:** sensory substitution, neuroprosthesis, peripheral neuropathy, fMRI, brain networks, functional connectivity

## Abstract

Foot sole somatosensory impairment associated with peripheral neuropathy (PN) is prevalent and a strong independent risk factor for gait disturbance and falls in older adults. Walkasins, a lower-limb sensory prosthesis, has been shown to improve gait and mobility in people with PN by providing afferent input related to foot sole pressure distributions *via* lower-leg mechanical tactile stimulation. Given that gait and mobility are regulated by sensorimotor and cognitive brain networks, it is plausible improvements in gait and mobility from wearing the Walkasins may be associated with elicited neuroplastic changes in the brain. As such, this study aimed to examine changes in brain network connectivity after 26 weeks of daily use of the prosthesis among individuals with diagnosed PN and balance problems. In this exploratory investigation, assessments of participant characteristics, Functional Gait Assessment (FGA), and resting-state functional magnetic resonance imaging were completed at study baseline and 26 weeks follow-up. We found that among those who have completed the study (*N* = 8; mean age 73.7 years) we observed a five-point improvement in FGA performance as well as significant changes in network connectivity over the 26 weeks that were correlated with improved FGA performance. Specifically, greater improvement in FGA score over 26 weeks was associated with increased connectivity within the Default Mode Network (DMN; *p* < 0.01), the Somatosensory Network (SMN; *p* < 0.01), and the Frontoparietal Network (FPN; *p* < 0.01). FGA improvement was also correlated with increased connectivity between the DMN and the FPN (*p* < 0.01), and decreased connectivity between the SMN and both the FPN (*p* < 0.01) and cerebellum (*p* < 0.01). These findings suggest that 26 weeks of daily use of the Walkasins device may provide beneficial neural modulatory changes in brain network connectivity *via* the sensory replacement stimulation that are relevant to gait improvements among older adults with PN.

## Introduction

Peripheral neuropathy (PN) is characterized by progressive peripheral nerve damage that typically starts in the distal extremities and progresses proximally. It affects more than 30 million individuals within the United States alone (Foundation for Peripheral Neuropathy, 2022). PN-related impairment in foot sole somatosensation is a strong, independent risk factor for postural instability (Meyer et al., 2004), mobility decline (Lipsitz et al., 2018), and falls (Richardson and Hurvitz, 1995). Recent evidence suggests that sensory substitution may be used to improve gait and balance in individuals with PN (Koehler-McNicholas et al., 2019; Oddsson et al., 2020). In particular, Walkasins ® (RxFunction Inc., MN, United States) is a wearable lower limb sensory prosthesis comprised of a foot pad embedded with pressure sensors that is inserted under each foot inside the shoes. This module is connected to a leg unit containing embedded control electronics, a rechargeable battery and four tactile stimulators placed over the skin around the lower calf. These stimulators provide near real-time mechanical tactile stimuli related to the center of pressure, calculated from the foot sole pressure signals. These new spatially distinct and directional specific balance stimuli are provided to the anterior–posterior and medial-lateral surfaces of the user’s calves. In a within-subject randomized cross-over study, Koehler-McNicholas et al. (2019) tested 31 individuals with PN and demonstrated that wearing the Walkasins resulted in near immediate improvements in the Functional Gait Assessment (FGA). Results of a subsequent clinical trial of 45 individuals with PN further indicated that daily use of the Walkasins device for 10 consecutive weeks was associated with clinically meaningful improvements in both gait function and mobility (Oddsson et al., 2020). After 26 weeks of use, these improvements were sustained and associated with a 43% decrease in falls in individuals who had fallen in the 6 months prior to participation in the study (Oddsson et al., 2022).

Gait and mobility are regulated by complex control systems that depend upon numerous sensorimotor and cognitive brain networks to process, integrate, and utilize sensory information for control of both automatic and volitional movements (Peterka, 2002; Woollacott and Shumway-Cook, 2002). Few studies have examined neuroimaging correlates of postural control, for which majority of the literature focused on Parkinson’s population (Massa et al., 2019; Kaut et al., 2020; Pasman et al., 2021). Evidence on functional connectivity, however, are scarce and equivocal. Resting-state functional MRI studies reported both increased and decreased regional connectivity comparing healthy older adults and their counterparts diagnosed with Parkinson’s disease with/without a history of falls (Kaut et al., 2020; Pasman et al., 2021). One study suggests that compared with health controls, individuals with Parkinson’s disease exhibited increased connectivity between regions of the Somatosensory Network (SMN) and the Frontoparietal Network (FPN) of the brain during simulated balance perturbations (Pasman et al., 2021). Another found that compared with healthy controls, those with Parkinson’s disease and history of falls showed increased connectivity within the cerebellum; but those with Parkinson’s disease and no history of falls showed decreased connectivity within nodes of the Default Mode Network (DMN) (Kaut et al., 2020). With regards to aspects of gait, our group have previously demonstrated that the aberrant resting-state functional connectivity of the SMN (Hsu et al., 2018) and FPN (Lo et al., 2017; Hsu et al., 2018) was correlated with slow gait speed among older individuals with possible signs of cognitive impairment; whereas connectivity of the DMN was linked to greater variability (i.e., less steadiness) during gait cycles (Lo et al., 2017, 2021). We therefore contend that observed functional improvements associated with sensory substitution in general, and with the Walkasins device in particular, arise at least in part from brain-level utilization of the novel source of sensory mechanical tactile stimuli.

Given the concept of neuroplasticity, we therefore hypothesized that extended use of the Walkasins sensory substitution device would be associated with measurable changes in brain function within and between networks involved in gait and postural control. To test this hypothesis, we conducted a pilot study as part of the larger clinical trial mentioned above (Oddsson et al., 2020), in which interested and eligible participants of one study site completed an optional resting-state MRI before and after extended daily use of the Walkasins device. Primary analyses focused on the identification of changes in the strength of resting-state functional connectivity—within three predetermined large-scale networks with putative involvement in the control of gait and mobility.

## Materials and methods

### Study design and participants characteristics

The walk2Wellness Trial (NCT03538756) was a multi-site study (Baylor College of Medicine, Houston, TX; Hebrew SeniorLife, Boston, MA, United States; VA Medical Center, Minneapolis, MN, United States; and M Health Fairview, Minneapolis, MN, United States) designed to investigate the effects of habitual Walkasins usage on gait, balance, and mobility over 1 year in individuals with PN. Individuals were eligible if they were between the ages of 18 and 90 years and presented with: (1) formal diagnosis of sensory peripheral neuropathy; (2) subjective balance complaints, (3) objective functional deficits defined by a Functional Gait Assessment score < 23/30 (the criteria for high fall risk), and (4) the ability to detect the four distinct mechanical tactile stimuli located on the anterior–posterior and medial-lateral surfaces of the lower leg provided by the Walkasins device.

Participants completed the screening assessment and if eligible, the baseline clinical assessment and MRI, both of which were repeated at 26 weeks. Participant demographics including age and sex were recorded. Additional participant characteristics included gait speed assessment and the Timed Up and Go Test (TUG) (Mathias et al., 1986). Briefly, gait speed was calculated as the time taken to ambulate 6 m in a 10-m walk test. The first and last 2 m of the 10-m walk were intended as buffers to account for acceleration/deceleration effects. A change in gait speed by 0.05 m/s is perceived as minimally clinically important difference (MCID) (Perera et al., 2006). The TUG requires research participants to rise from seated position on a chair, walk 4 m, pivot, then return to seated position while being timed throughout the entire process. The TUG has been validated and proven to be reliable in assessing general mobility of older adults (Podsiadlo and Richardson, 1991).

Enrolled participants at Hebrew SeniorLife, Boston, MA, United States site were offered the opportunity to partake in an optional MRI pilot study. Interested participants were included into this sub-study if they were eligible and able to complete a brain MRI at study baseline and 26-week follow-up. IRB-approved informed consent was provided prior to study participation. Though the COVID-19 pandemic limited recruitment, eight of 10 older adults recruited from the parent study at this site completed this MRI sub-study in full.

### Walkasins sensory prosthetic device

The participants were provided with the device at study baseline and instructed to wear the Walkasins device as much as possible throughout daily living activities over the 26 weeks. Walkasins is a novel sensory-prosthesis designed to replace lost foot-sole sensation by providing vibrotactile stimulation. The device consisted of a foot pad placed in the shoe integrated with pressure sensors, as well as a lower-leg unit that contains four motors (located at anterior, posterior, medial, and lateral sides above the ankle) which received inputs from the pressure sensors then provide gentle mechanical tactile stimuli to the lower legs to replace/substitute the absence of foot-sole sensation due to peripheral neuropathy (Supplementary Figure 1). Please refer to previous publication for more detail on the device (Koehler-McNicholas et al., 2019; Oddsson et al., 2020).

During the baseline assessment, study participants underwent a 10-min training session to gain familiarity with the device. The training involved standing (two-leg, tandem, and one-leg standing) as well as walking (straight, turning right and left) at normal and fast speed. Participants performed two rounds of the training protocol, with the device off and turned on, respectively. They were asked to pay particular attention to the tactile stimuli during all phases of the training session.

### Functional gait assessment

Functional Gait Assessment was assessed at both study baseline and at 26-weeks follow-up (Wrisley et al., 2004). The FGA is a 10-item test with a maximum score of 30 where higher score reflects greater function. The 10 items evaluate normal gait speed, change in gait speed, change in gait with head turn, change in gait with body pivot, stepping over obstacle, gait with narrow base of support, gait with eyes closed, walking backwards, and stair stepping. The FGA has been validated with good interrater and intra-rater reliability as well as internal consistency (Wrisley et al., 2004). An MCID of four points among community-dwelling older adults was established by previous study (Beninato et al., 2014).

### Neuroimaging

Resting-state functional magnetic resonance imaging (rs-fMRI) was completed at study baseline and 26-weeks follow-up on a 3 T Siemens TIM Trio scanner with 12-channel head coil. Imaging acquisition for the four rs-fMRI trials is as follows: TR = 800 ms, TE = 26 ms, FA = 90 degrees, 34 slices at 1.5 mm slice thickness, acquisition matrix = 64×64, and a total of 120 volumes. T1-weighted (T1w) high spatial resolution anatomical image was acquired with MPRAGE sequence with the following parameters: TR = 2.73 ms, TE = 3.31 ms, FA = 7 degrees, 128 slices at 1.3 mm thickness, acquisition matrix = 256×256. No particular instructions were provided to study participants during the session.

#### Network of interest selection

To-date, fMRI-derived neuroimaging correlates of gait, balance, and mobility in PN are unknown. As such, we examined three functional neural networks with putative roles in the neural processing of sensory inputs/motoric outputs pertaining to postural control and gait with observed links to complex control of mobility in older adults. These included the Default Mode Network (DMN), Somatosensory Network (SMN), and Fronto-Parietal Network (FPN) (Crockett et al., 2017; Lo et al., 2017; Hsu et al., 2018; Poole et al., 2019).

### Functional MRI and statistical analysis

Functional imaging processing was performed using a combination of FSL (FMRIB’s Software Library), MATLAB (Matrix Laboratory), and toolboxes within CONN (Whitfield-Gabrieli and Nieto-Castanon, 2012) as well as SPM (Statistical Parametric Mapping). Initial brain extraction of the high resolution T1w images was carried out *via* optimized Brain Extraction Tool (optiBET) (Lutkenhoff et al., 2014) followed by standard preprocessing pipeline including rigid body motion correction, spatial smoothing with a 6.0 mm Full-Width-Half-Maximum Gaussian kernel, co-registration of the functional and brain-extracted anatomical images to the common Montreal Neurological Institute (MNI) 2.0 mm standard template. CONN was used for a component-based noise reduction (CompCor) (Behzadi et al., 2007) that produced five noise components for each white matter and cerebral spinal fluid nuisance signals, which were subsequent regressed out from the time-series data. A band-pass filter of 0.008 Hz to 0.08 Hz was used to ensure confounding physiological signals of no interest were removed. Participant motion was captured as frame-wise displacement with a cut-off of <0.5 mm indicating excessive movement that was visually inspected by CLH.

Seed-based resting-state functional connectivity was computed *via* a regions-of-interest (ROI) approach for the DMN, SMN, and FPN. First-level (i.e., subject level) analysis yielded a ROI-to-ROI connectivity matrix for each study participant by calculating and transforming bivariate correlations between the networks of interest to normalized Fisher’s z correlation coefficients. Second-level analysis subsequently applied general linear model to examine connectivity patterns at each time-point across all study participant. FGA-related network connectivity maps were established *via* General Linear Model (GLM). Specifically, the mean-centered FGA score for each study participant as well as baseline outcome measures were included in the GLM as additional covariate during the computation. For the purpose of this exploratory analysis, all *p*-values reported are uncorrected *p*-values. Lastly, statistical brain connectivity maps were generated with a threshold at Z > 2.7 with a cluster significance threshold of *p* < 0.05.

## Results

### Participant characteristics and functional gait assessment

Participant characteristics and FGA performance are reported in Table 1. The average baseline age of participants was 72.7 (SD = 8.2) years. Participants exhibited an average of a 5.0 point [SD = 2.16; *t*(6) = −6.12, *p* < 0.001] increase (i.e., improvement) in FGA total score from baseline to the 26-week follow-up assessment. Notably, all participant exhibited a higher score on the FGA at the follow-up as compared to baseline. Average improvement in gait speed was 0.12 m/s [SD = 0.17; *t*(6) = −1.76, *p* = 0.13], which exceeded the MCID for gait speed (Perera et al., 2006). Average improvement in TUG was 1.10 s [SD = 1.79; *t*(6) = 1.84, *p* = 0.12].

**Table 1:** Participant characteristics (*N* = 8).

	Baseline	26-Weeks
	Mean (SD)	Mean (SD)
Characteristics		
Age (yrs)	73.7 (7.2)	–
Sex (M/F)	5/3	–
Gait speed (m/s)	0.98 (0.10)	1.10 (0.19)
Timed up and go test (sec)	12.13 (1.12)	11.03 (1.79)
Primary outcome measure		
Functional gait assessment (max 30)	14.71 (2.22)	19.71 (1.50)

### Correlation between FGA performance and resting-state fMRI at baseline

**Figure 1:**
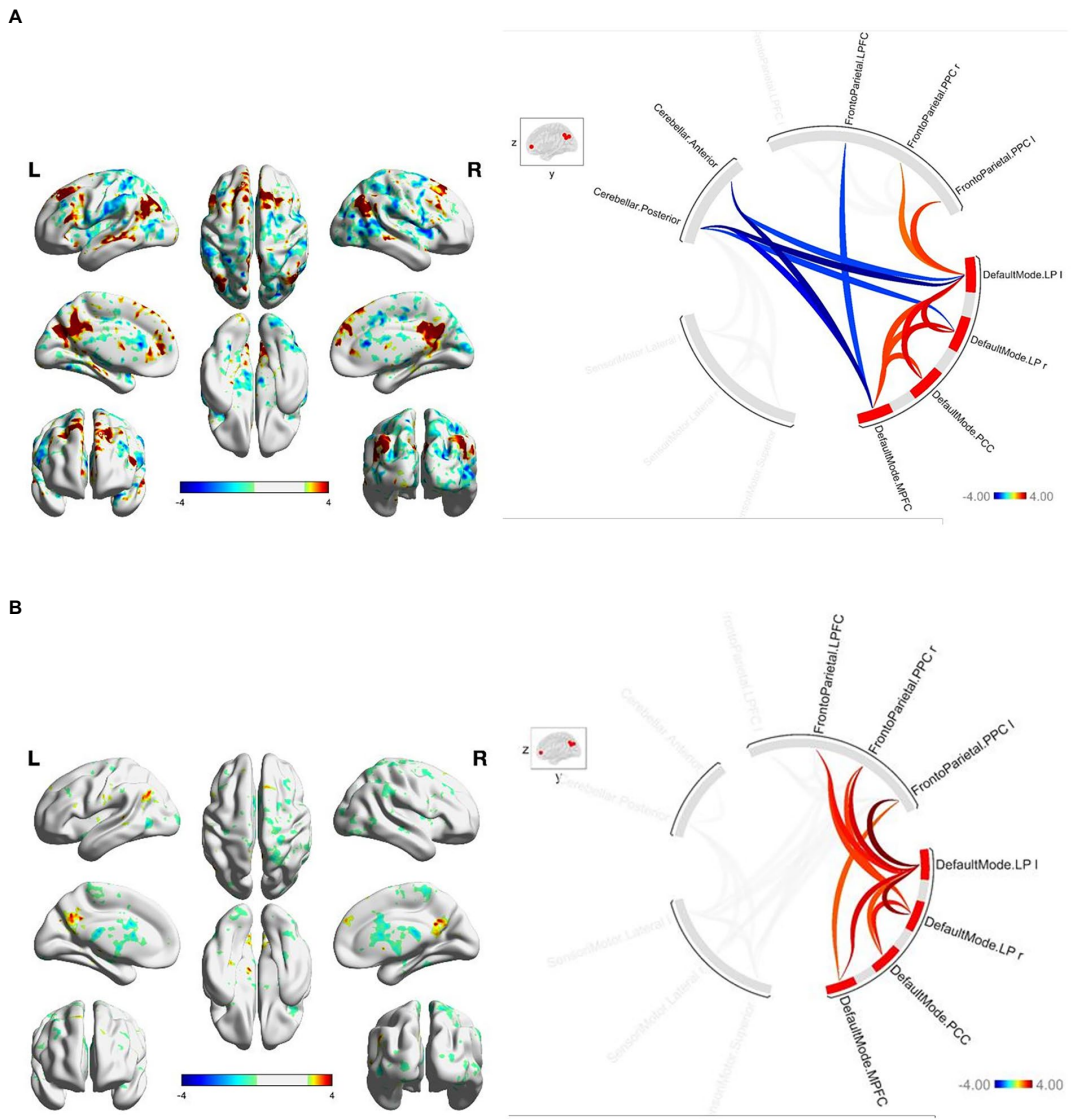
**(A)** FGA-related DMN connectivity at baseline. For connectome ring: bottom right quadrant contains nodes of the DMN, top right quadrant contains nodes of the FPN, the top left and bottom left quadrants contain nodes of the cerebellum and SMN. At baselight greater FGA score was associated with: greater within-network connectivity within the DMN (uncorrected *p* < 0.01); greater connectivity between the DMN and FPN (uncorrected *p* < 0.01); lower connectivity between the DMN and cerebellum (uncorrected *p* = 0.02). **(B)** FGA-related DMN connectivity over 26-weeks (26 week – baseline). For connectome ring: bottom right quadrant contains nodes of the DMN, top right quadrant contains nodes of the FPN, the top left and bottom left quadrants contain nodes of the cerebellum and SMN. At 26-weeks greater FGA score was correlated with: greater within-network connectivity within the DMN (uncorrected *p* < 0.01); greater connectivity between the DMN and FPN (uncorrected *p* < 0.01); Statistical brain maps were generated *via* general linear model (Z > 2.7, *p* < 0.05 FWE corrected).

**Figure 2:**
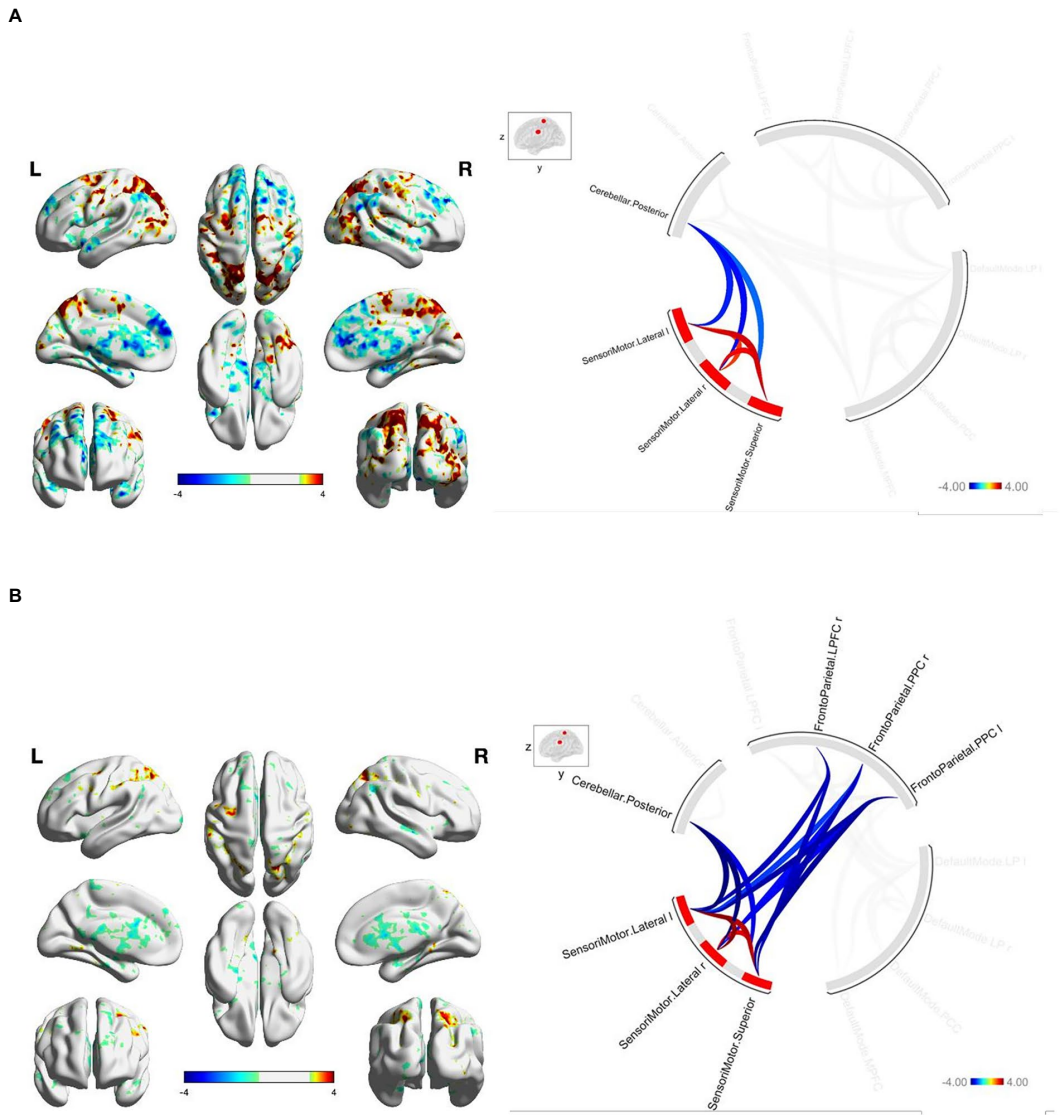
**(A)** FGA-related SMN connectivity at baseline. For connectome ring: bottom right quadrant contains nodes of the DMN, top right quadrant contains nodes of the FPN, the top left and bottom left quadrants contain nodes of the cerebellum and SMN. At baseline greater FGA score associated with: greater within-network connectivity within the SMN (uncorrected *p* < 0.01); lower connectivity between the SMN and cerebellum (uncorrected *p* < 0.01). **(B)** FGA-related SMN connectivity over 26-weeks (26 week – baseline). For connectome ring: bottom right quadrant contains nodes of the DMN, top right quadrant contains nodes of the FPN, the top left and bottom left quadrants contain nodes of the cerebellum and SMN. At 26-Weeks greater FGA score was correlated with: greater within-network connectivity within the SMN (uncorrected *p* < 0.01); lower connectivity between the SMN and cerebellum (uncorrected *p* < 0.01); lower connectivity between the SMN and FPN (uncorrected *p* < 0.01); Statistical brain maps were generated *via* general linear model (Z > 2.7, *p* < 0.05 FWE corrected).

**Figure3:**
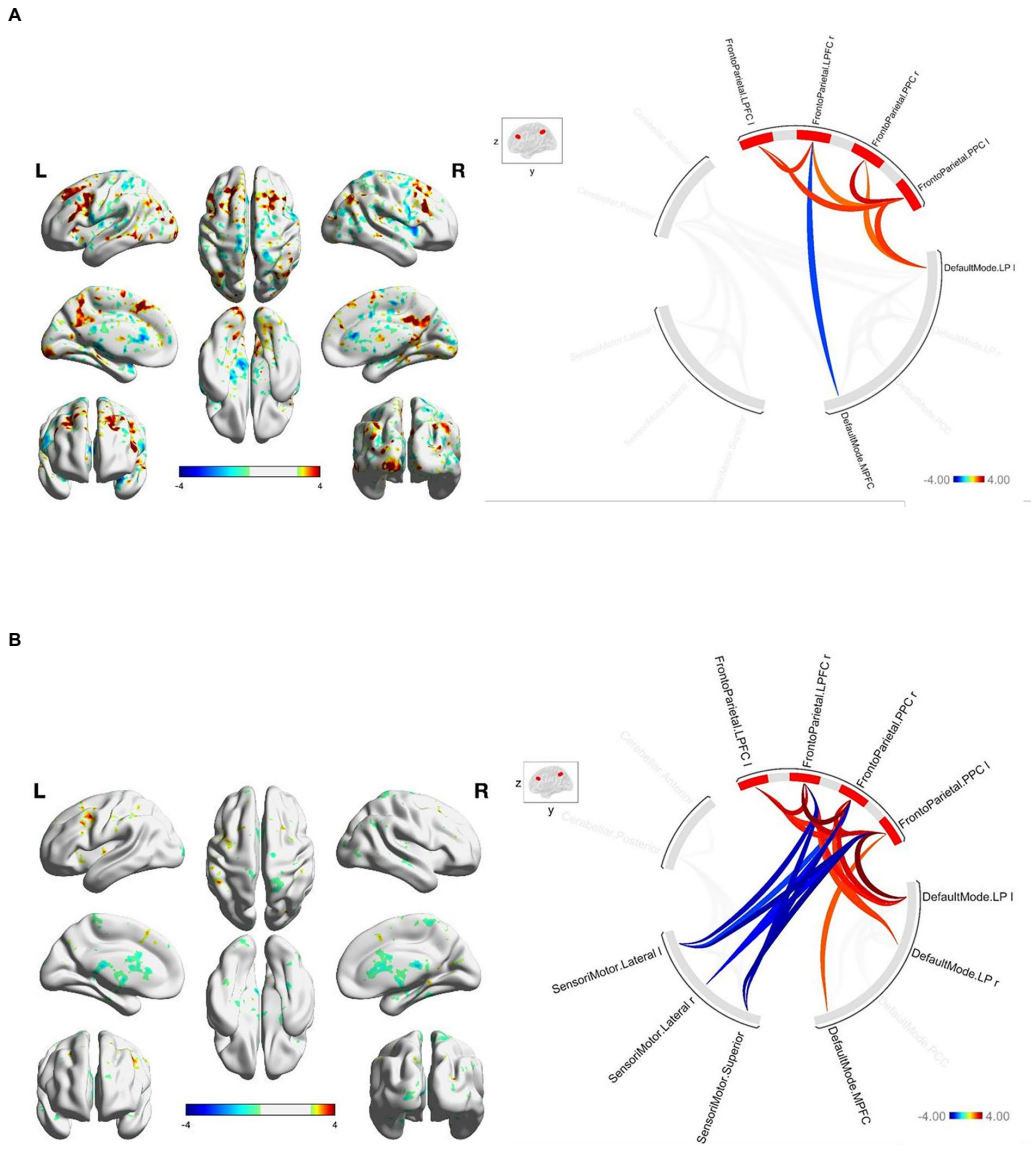
**(A)** FGA-related FPN connectivity at baseline. For connectome ring: bottom right quadrant contains nodes of the DMN, top right quadrant contains nodes of the FPN, the top left and bottom left quadrants contain nodes of the cerebellum and SMN. At baseline greater FGA score associated with: greater within-network connectivity within the FPN (uncorrected *p* < 0.01); greater connectivity between the DMN and FPN (uncorrected *p* < 0.01). **(B)** FGA-related FPN connectivity over 26-weeks (26 week – baseline). For connectome ring: bottom right quadrant contains nodes of the DMN, top right quadrant contains nodes of the FPN, the top left and bottom left quadrants contain nodes of the cerebellum and SMN. At 26-weeks greater FGA score was correlated with: greater within-network connectivity within the SMN and FPN (uncorrected *p* < 0.01); greater connectivity between the DMN and FPN (uncorrected *p* < 0.01); Statistical brain maps were generated *via* general linear model (Z > 2.7, *p* < 0.05 FWE corrected).

At baseline, FGA performance was significantly correlated with both within-network and between-network resting-state functional connectivity of the three networks included as primary seeds in the fMRI analysis. Specifically, greater FGA score was associated with greater within-network connectivity of the DMN (uncorrected *p* < 0.01; Figure 1A), SMN (uncorrected *p* = 0.01; Figure 2A), and FPN (uncorrected *p* < 0.01; Figure 3A). In terms of between-network associations, we observed that greater FGA performance was associated with greater connectivity between the DMN and FPN (uncorrected *p <* 0.01; Figures 1A, 3A), lower connectivity between the SMN and cerebellum (uncorrected p < 0.01, Figure 2A), as well as lower connectivity between the DMN and cerebellum (uncorrected *p* = 0.02; Figure 1A).

### Correlation between improvement in FGA performance and changes in resting-state fMRI over 26  weeks

We observed significant changes in network connectivity over the 26 weeks that were correlated with FGA performance. In particular, greater improvement in FGA score over 26 weeks was associated with increased connectivity *within* the DMN (uncorrected *p* < 0.01; Figure 1B), the SMN (uncorrected *p* < 0.01; Figure 2B), and the FPN (uncorrected *p* < 0.01; Figure 3B).

The magnitude of FGA improvement was also correlated with increased connectivity *between* the DMN and nodes of the FPN (uncorrected *p* < 0.01; Figures 1B, 3B), as well as decreased connectivity *between* the SMN and nodes of both the FPN (uncorrected *p* < 0.01; Figure 3B) and cerebellum (uncorrected *p* < 0.01; Figure 2B).

## Discussion

The current exploratory analysis examined rs-fMRI data collected from a subset of participants in the walk2Wellness Trial. At baseline, among the eight study participants, greater FGA performance was associated with greater within-network connectivity in the DMN, SMN and FPN. It was also correlated with greater between-network connectivity between the DMN and FPN, and lower connectivity between cerebellum and both the SMN and DMN. Participants demonstrated improved FGA performance after 26 weeks and most notably, the improvement in FGA performance was significantly associated with increased within-network connectivity of the DMN, SMN and FPN; as well as with increased between-network connectivity between the DMN and FPN, and decreased between-network connectivity between the SMN and both the cerebellum and FPN. While this study may not be statistically powered to fully elucidate underlying changes in resting-state network associated with FGA performance, these results may be preliminary indications of neural modulatory capabilities of prosthetic sensory replacement provided by Walkasins after extended application.

### Improved FGA performance and change in within-network connectivity

Improvement in FGA performance over time was significantly correlated with greater within-network connectivity in the DMN, SMN, and FPN. Evidence in the literature has established presence of neuroplasticity that even among older adults, intervention-induced changes in functional network connectivity can be observed (Voss et al., 2010; Won et al., 2021). Hence, elevated within-network connectivity can be perceived as a shift towards greater modularity in functional neural network organization, which has been previously posited as an advantageous attribute associated with better cognitive functioning (Wang et al., 2020; Gu et al., 2022), as well as resiliency to dementia (Ewers et al., 2021). In particular, greater within-network connectivity of the DMN has been demonstrated to be linked with better memory (Vidal-Pineiro et al., 2014) and executive function performance (Andrews-Hanna et al., 2007) in healthy older adults. Similarly, lower SMN within-network connectivity was reportedly found among older adults with mild cognitive impairment and Alzheimer’s disease (Wang et al., 2015). Greater local efficiency of the SMN was also suggested to be involved in better gait stability among older adults (Di Scala et al., 2019). The FPN, on the other hand, is intricately linked with cognitive control and executive functions (Ptak, 2012). Greater FPN within-network connectivity was found to mitigate the effect of white matter lesions on executive dysfunction and is relevant to greater cognitive reserve (Chen et al., 2022). In light of the well-recognized bidirectional relationship between cognition and mobility (Camicioli, 2017), the merits of modular network organization may be extrapolated to mobility and postural control.

Our exploratory investigation demonstrated that after 26 weeks of exposure to Walkasins, older adults with PN may be able to maintain modular configuration across all three functional neural networks, which likely underlies the exhibited improvements in FGA performance. As such, at the conceptual level, our findings support the notion that greater modular framework in the brain secondary to peripheral sensory stimulation *via* Walkasins may be clinically beneficial. Albeit beyond the scope of the current study, the sensory tactile stimuli provided by Walkasins could also indirectly increase physical activity among the participants. Greater physical activity level, independently or in combination with sensory stimulation, may have led to the observed changes in neural network connectivity. Nevertheless, future research with larger sample size and a clinically matched control-group with extensive physical activity measurements would be required to confirm this proposition.

### Improved FGA performance and change in between-network connectivity

Across the 26 weeks, we observed that greater improvement in FGA performance was significantly correlated with greater increase in connectivity between DMN and FPN, decreased connectivity between SMN and cerebellum as well as decreased between-network connectivity between the SMN and FPN. Notably, the current view of increased between network connectivity between the DMN and FPN has been largely unfavorable, where among healthy individuals, an anti-correlation is typically exhibited (Greicius et al., 2003; Fox et al., 2005). The canonical thought is that this intrinsic dichotomy between the networks represents an anti-synchronized behavior between task-positive (i.e., goal-oriented) and task-negative networks, reflecting a relationship fluctuating between introspection, self-referential thoughts contributed by the DMN versus extrospection, attention-oriented cognitive state contributed by the FPN (Buckner et al., 2008). At the moment we cannot be certain of the rationale behind why improved FGA performance was associated with a Walkasins-induced increase in functional connectivity between DMN and FPN. However, research from Vatansever et al. (2015, 2017, 2018) suggest that the DMN may not be entirely dissociated from higher order cognitively demanding tasks, but rather, it may be involved in more automated behaviors that require rapid responses. As such, one potential postulate of our results is that among older individuals with PN, stimulations provided by Walkasins may have enhanced this particular aspect of the DMN, where components of the FGA are reliant on quick, automated responses. Hence, the increase in between-network connectivity between DMN and FPN may reflect a state of augmented automatic information processing from the DMN in conjunction with goal-oriented cognitive processing from the FPN.

The observed decrease in SMN-FPN between-network connectivity may also reflect greater modularity in neural networks, which is in accordance to our within-network findings, further strengthening the idea that sustained sensory replacement stimulation provided by Walkasins may have reinforced modular neural network architecture. Potentially, the Walkasins-induced decrease in connectivity between SMN-FPN that we observed may not be entirely counterintuitive. The SMN and FPN between-network coupling has been previously found to play a role in cognitive and mobility function (Hsu et al., 2018). Findings from this cross-sectional investigation showed among older individuals with mild cognitive impairments, slower gait speed was significantly correlated with greater connectivity between regions of the SMN and FPN (Hsu et al., 2018).

Similarly, we found that using Walkasins for 26 weeks introduced a decrease in between-network connectivity between SMN and cerebellum. While the exact extent and role of cerebellum functional coupling is not fully understood, a previous study reported that there is little to no cerebellar connectivity to SMN (Habas et al., 2009), suggesting these two networks may have distinctively segregated functional roles. Past literature has linked cerebellum connectivity to cognitive function (Habas et al., 2009; Pagen et al., 2020). Of particular interest, aligning with our findings of potential benefits of decreased connectivity to the cerebellum was associated with improved FGA performance, one previous study has demonstrated that compared with healthy older adults, their counterparts with mild cognitive impairment exhibited lower anti-correlation (i.e., increased functional connectivity) between DMN and cerebellum - which was correlated with poorer memory performance (Pagen et al., 2020). Lower anti-correlation (i.e., increased functional connectivity) was proposed to negatively impact modularity of the cerebellum in its role on cognitive functioning. Albeit we did not replicate similar relationship between DMN and cerebellum in our analysis (may be due to differences in study participant populations, characteristics, etc.), our findings of decreased connectivity between SMN and cerebellum may be subject to a similar logic. However, it is important to note that most fMRI protocols are less well-equipped to properly examine cerebellar structures, hence, future studies will be necessary to confirm whether it would be beneficial to exhibit decreased connectivity between SMN and the cerebellum.

There are several limitations to be considered. First, given the sample size and exploratory nature of this study, our findings and the discussed underlying functional connectivity patterns should be cautiously interpreted. Future investigations with a larger sample size and clinically-matched control group would be required to confirm our results. Increasing the overall sample size with the addition of a control group would also enable more comprehensive statistical analyses and a more exhaustive list of networks/regions of interest to better understand the neural mechanistic pathways underpinning the marked functional improvements after using Walkasins. A larger randomized controlled trial would also allow long-term investigations of plausible prolonged neural and physical benefits of the Walkasins. Lastly, we cannot rule out potential confounding effects of neural degenerative pathologies or cognitive impairments.

## Conclusion

To conclude, our novel exploration demonstrated that 26 weeks of daily use of the Walkasins prosthetic device appeared to be an effective intervention strategy for improving postural control and gait function among older adults with PN. Despite the small sample size of this exploratory study, we observed noticeable alterations to functional neural network connectivity patterns associated with improved postural control. This may be indicative of probable beneficial neural modulatory effects *via* the sensory replacement stimulation provided by the Walkasins sensory prosthesis.

## Data availability statement

The raw data supporting the conclusions of this article will be made available by the authors, without undue reservation.

## Ethics statement

The studies involving human participants were reviewed and approved by Advarra IRB (formerly Quorum Review IRB), serving as the Institutional Review Board (IRB) of record. The study is registered on ClinicalTrials.gov (#NCT03538756). The patients/participants provided their written informed consent to participate in this study.

## Author contributions

CLH contributed to all aspects of this manuscript, including data analysis, data interpretation, and writing and editing of the manuscript. BM, II, LO, and LL contributed to reviewing, writing, editing, and approval of the manuscript. All authors contributed to the article and approved the submitted version.

## Funding

CLH is supported by the Canadian Institute for Health Research. BM is supported in part by NIH grants R01 AG059089 and R21 AG064575. LL holds the Irving and Edyth S. Usen and Family Chair in Geriatric Medicine at Hebrew SeniorLife. A grant from RxFunction supported data collection. Walkasins were provided by RxFunction.

## Conflict of interest

Walkasins was provided by RxFunction. LO is co-inventor, co-founder, shareholder, and a member of the Board of Directors of RxFunction, Inc.

The remaining authors declare that the research was conducted in the absence of any commercial or financial relationships that could be construed as a potential conflict of interest.

## Publisher’s note

All claims expressed in this article are solely those of the authors and do not necessarily represent those of their affiliated organizations, or those of the publisher, the editors and the reviewers. Any product that may be evaluated in this article, or claim that may be made by its manufacturer, is not guaranteed or endorsed by the publisher.

## Supplementary material

The Supplementary material for this article can be found online at: https://www.frontiersin.org/articles/10.3389/fnagi.2022.1027242/full#supplementary-material
