# Supplementary material for: Six month lower-leg mechanical tactile sensory stimulation alters functional network connectivity associated with improved gait in older adults with peripheral neuropathy – A pilot study
Source: Front Aging Neurosci. 2022 Nov 3;14:1027242. doi: 10.3389/fnagi.2022.1027242 (PMC9669982; doi:10.3389/fnagi.2022.1027242)
Supplement: Supplementary file 1 [file Image_1.pdf]

Supplementary Figure 1. Walkasins Device

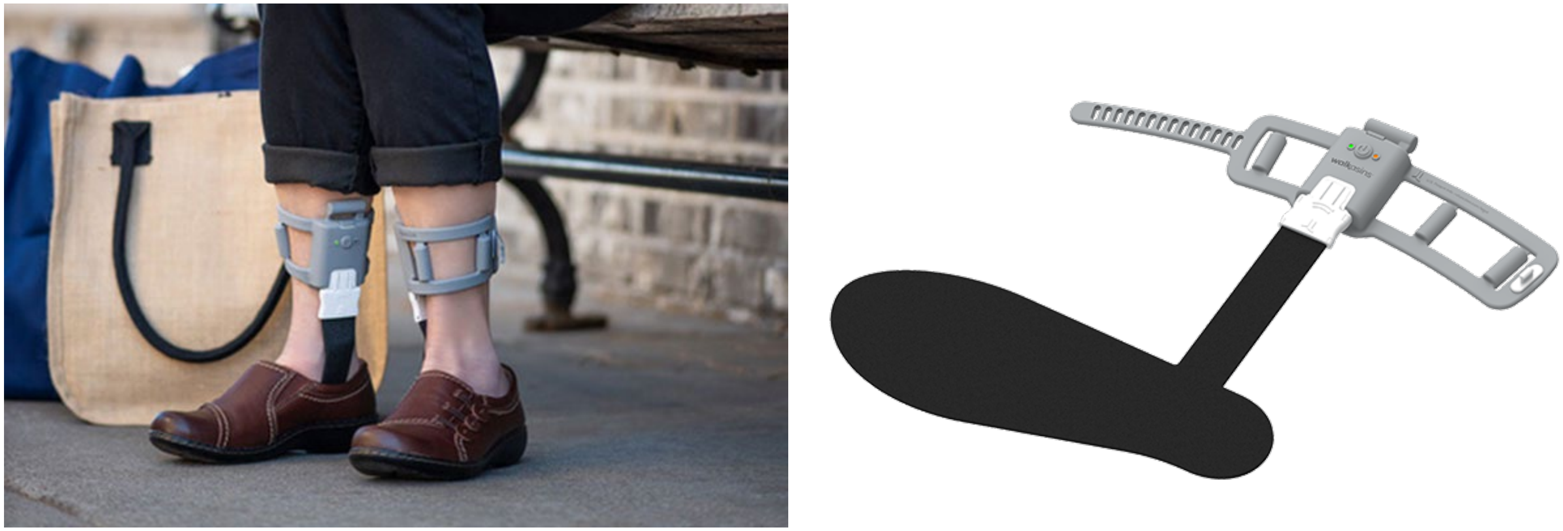

Walkasins sensory prosthesis and its two components. Foot pad is placed inside the bottom of the shoe and the leg unit is placed around the lower calves.
